# Pro-atrial natriuretic peptide is a prognostic marker in sepsis, similar to the APACHE II score: an observational study

**DOI:** 10.1186/cc3015

**Published:** 2004-12-17

**Authors:** Nils G Morgenthaler, Joachim Struck, Mirjam Christ-Crain, Andreas Bergmann, Beat Müller

**Affiliations:** 1 Research Department, BRAHMS AG, Biotechnology Center, Hennigsdorf/Berlin, Germany; 2 Department of Internal Medicine, University Hospital, Basel, Switzerland

**Keywords:** biomarkers, diagnosis, sepsis, therapy monitoring

## Abstract

**Introduction:**

Additional biomarkers in sepsis are needed to tackle the challenges of determining prognosis and optimizing selection of high-risk patients for application of therapy. In the present study, conducted in a cohort of medical intensive care unit patients, our aim was to compare the prognostic value of mid-regional pro-atrial natriuretic peptide (ANP) levels with those of other biomarkers and physiological scores.

**Methods:**

Blood samples obtained in a prospective observational study conducted in 101 consecutive critically ill patients admitted to the intensive care unit were analyzed. The prognostic value of pro-ANP levels was compared with that of the Acute Physiology and Chronic Health Evaluation (APACHE) II score and with those of various biomarkers (i.e. C-reactive protein, IL-6 and procalcitonin). Mid-regional pro-ANP was detected in EDTA plasma from all patients using a new sandwich immunoassay.

**Results:**

On admission, 53 patients had sepsis, severe sepsis, or septic shock, and 68 had systemic inflammatory response syndrome. The median pro-ANP value in the survivors was 194 pmol/l (range 20–2000 pmol/l), which was significantly lower than in the nonsurvivors (median 853.0 pmol/l, range 100–2000 pmol/l; *P *< 0.001). On the day of admission, pro-ANP levels, but not levels of other biomarkers, were significantly higher in surviving than in nonsurviving sepsis patients (*P *= 0.001). In a receiver operating characteristic curve analysis for the survival of patients with sepsis, the area under the curve (AUC) for pro-ANP was 0.88, which was significantly greater than the AUCs for procalcitonin and C-reactive protein, and similar to the AUC for the APACHE II score.

**Conclusion:**

Pro-ANP appears to be a valuable tool for individual risk assessment in sepsis patients and for stratification of high-risk patients in future intervention trials. Further studies are needed to validate our results.

## Introduction

Affecting about 700,000 people annually, sepsis accounts for 210,000 deaths each year in the USA, and both of these figures are likely to increase [1,2]. Sepsis is not an homogenous disease; rather, it is a complex clinical syndrome with distinct immunological features [3,4]. The ambiguity of clinical findings and unclear risk stratification in sepsis have been major problems in sepsis intervention trials [5]. The effectiveness of anti-inflammatory treatment correlates with risk for death and severity of disease [6]. Thus, the prognosis of a septic patient may contribute significantly to the success of any intervention [5]. Within this context, there is need for biomarkers to tackle the challenges of sepsis monitoring and treatment [7].

Members of the natriuretic peptide family are established markers of congestive heart failure [8-10]. Defending against hypertension and salt and water retention, they antagonize the renin–angiotensin–aldosterone system, including effects on renal tubule sodium reabsorption, vascular tone and cell growth. Atrial natriuretic peptide (ANP) is predominantly produced in the atrium of the heart and comprises 98% of natriuretic peptides in the circulation [11]. Recently, both ANP and pro-ANP have attracted interest as new markers in the field of sepsis [12-16]. Mature ANP is derived from carboxyl-terminal amino acids 99–126 of the prohormone (pro-ANP), which is 126 amino acids in length [11]. The amino-terminal portion of pro-ANP (termed NT-pro-ANP, or pro-ANP_1–98_) is secreted at the same molar ratio as ANP. Because it has a much longer half-life than has mature ANP, it has been suggested that pro-ANP_1–98 _is a more reliable analyte [17]. However, results from various competitive immunoassays and high-performance liquid chromatography analyses indicate that pro-ANP_1–98 _may be subject to further fragmentation [18,19]. Consequently, sandwich immunoassays for pro-ANP_1–98 _might underestimate actual levels of pro-ANP, and immunoassays for measurement of mid-regional pro-ANP may have an advantage [20].

In the present study we aimed to evaluate the prognostic value of mid-regional pro-ANP levels in a well defined cohort of medical intensive care unit (ICU) patients as compared with those of other biomarkers (i.e. IL-6, C-reactive protein [CRP] and procalcitonin [PCT]) and a physiological score (Acute Physiology and Chronic Health Evaluation [APACHE] II).

## Methods

### Patients

In the present study we evaluated plasma samples from a cohort of 101 consecutive critically ill patients admitted to the medical ICU of the University Hospital of Basel, Switzerland. The primary end-point of this study was the prognostic value of endocrine dysfunction in critically ill patients ('PEDCRIP' study). The characteristics of the study population, study design, diagnostic criteria and levels of various markers of inflammation and infection were reported in detail elsewhere [21-24]. Briefly, over a 9-month period 101 consecutive patients, including neutropenic and immunosuppressed patients, admitted to the medical ICU were included. Patients were followed until hospital discharge or death.

Data were collected on admission (i.e. during the first 24 hours), on day 2, and on the day of discharge from the ICU or on the day of death. At these time points (a total of 276 plasma samples), patients were either very sick or in a stable condition and ready for discharge to a medical ward, respectively. In patients who died within 24 hours after admission, only data from admission were collected (*n *= 5). Vital signs, clinical status and severity of disease parameters (APACHE II score) were assessed daily. The APACHE II score was calculated by means of maximal daily deviations of 12 physiological variables from normal plus correction for age and various chronic illnesses. A pulmonary artery catheter was not routinely inserted. When feasible, consent was obtained from conscious patients before enrolment; otherwise, consent was obtained from the next of kin. The study protocol had been granted approval by the hospital institute's ethical review board.

Patients were classified at the time of blood collection into those with systemic inflammatory response syndrome (SIRS), sepsis, severe sepsis and septic shock, which were defined according to international criteria [25,26]. Infection was diagnosed according to standardized criteria or, in case of uncertainty, by an infectious disease specialist. This was done retrospectively on the basis of review of complete patient charts, results of microbiological cultures, chest radiographs and, when available, autopsy reports. An isolated micro-organism was considered to be pathogenic if it was identified within a 24-hour period before or after the onset of the systemic response. Colonization with bacteria (e.g. in a patient with a bladder catheter but without leucocyturia) or positive blood cultures at autopsy were disregarded. Microbiological tests were requested and antibiotic therapy prescribed by physicians on duty according to the usual practice, without interference from the research team.

Although optimal fluid resuscitation was done in the initial treatment phase in all patients, 31% of septic patients needed additional treatment with intravenous noradrenaline (norepinephrine). The mean dose of noradrenaline on admission was 8.7 ± 12.1 μg/min, on day 2 it was 10.1 ± 10.9 μg/min and on the day of discharge/death it was 47.2 ± 35.2 μg/min (*P *< 0.001). Nonsurvivors from severe sepsis and septic shock needed higher doses of noradrenaline than did survivors (5.7 ± 7.8 μg/min versus 30.5 ± 28.1 μg/min; *P *< 0.001). Overall, 23 of the 101 patients died (22.8%). The majority of patients who died suffered from multiple organ failure (56.5%), defined as failure of two or more vital organs.

### Assays

Results of the routine blood analyses (i.e. complete blood count, serum chemistry including CRP, blood gas analyses) were known and recorded. Blood was obtained from an indwelling arterial or venous catheter. Plasma was separated from the blood samples at the time of blood draw and frozen at -70°C until assayed. Measurement was done in a blinded manner as a batch analysis.

Mid-regional pro-ANP (epitopes covering amino acids 53–90) was detected in EDTA plasma from all patients with a new sandwich immunoassay (BRAHMS Seristra^® ^LIA; BRAHMS AG, Hennigsdorf/Berlin, Germany), as described in detail elsewhere [20]. As a modification to the published assay, the calibration was changed from a synthetic peptide to pro-ANP in human serum. This modification to the initial description increased the precision and dynamic (i.e. signal to noise ratio) of the assay, and allowed measurement of pro-ANP in serum and plasma (with EDTA, heparin, or citrate). Briefly, patient samples (1:40 dilution of 5 μl plasma in incubation buffer) or standards were added in duplicate to antibody-coated tubes (directed at pro-ANP peptide 73–90) and incubated for 30 min at room temperature. After five washings with 1 ml washing buffer, 200 μl tracer was added, containing acridinium ester-labelled anti-pro-ANP antibody (directed at peptide 53–72), followed by 30 min incubation at room temperature. Tubes were washed three times with 1 ml washing buffer, and detection was performed in a luminometer (1 s detection time per sample). Relative light units of the chemiluminescence assay were expressed in pmol/L pro-ANP, as calculated from a calibration curve (4–1800 pmol/l) that was included in every analytical run. The lower detection limit of the assay is 4.3 pmol/l and the functional sensitivity of the assay (interassay coefficient of variation <20 %) is 11 pmol/L pro-ANP. The 97.5th percentile in 325 healthy individuals was 163.9 pmol/l (median 45 pmol/l), with no difference between sexes [20].

PCT was measured using the LUMITest^® ^PCT (BRAHMS AG), following the manufacturer's instructions. CRP was determined using en enzyme immunoassay (EMIT; Merck Diagnostica, Zurich, Switzerland). A serum level greater than 5 mg/l was considered abnormally elevated. Serum IL-6 concentrations were measured using a commercially available quantitative sandwich enzyme immunoassay (Pelikine Compact™; CLB, Amsterdam, The Netherlands), with a limit of detection at 0.6 ng/l.

### Statistical analysis

Data in the text are expressed as mean ± standard deviation. Frequency comparison was done by χ^2 ^test. Two-group comparisons were performed using the Mann–Whitney U-test. For multigroup comparisons, Kruskal–Wallis one-way analysis of variance was used with Dunn's post-test evaluation. Levels that were nondetectable were assigned a value equal to the lower limit of detection for the assay. All testing was two-tailed, and *P *< 0.05 was considered statistically significant. Correlation analyses were performed by using Spearman rank correlation.

## Results

### Descriptive characteristics of the patients

The mean age of the 101 patients (55 men and 46 women) included in the study was 57 ± 15 years (range 23–86 years) and the mean APACHE II score on admission was 22 ± 8. The median length of stay in the medical ICU was 4 days (range 0.2–60 days) and the mortality rate was 23%. More detailed baseline characteristics of the study population are described elsewhere [21]; however, to allow better understanding of the study results, the principal diagnoses of patients are summarized in Table 1 and the sites of infection in Table 2. Sepsis was diagnosed in 58% of patients (on admission in 53 patients; five additional patients developed sepsis during their stay in the ICU). The principal site of infection was the lung (Table 2). In 38 (66%) of the 58 patients with infections, the responsible micro-organism was identified and 14 patients (24%) had bacteraemia. There was no difference in mortality between patients with and those without infection. Of the 53 patients admitted with sepsis, severe sepsis, or septic shock, 13 (25%) died; 10 (21%) of the 48 patients without infection on admission died.

### Pro-atrial natriuretic peptide and severity of the disease

**Figure 1:**
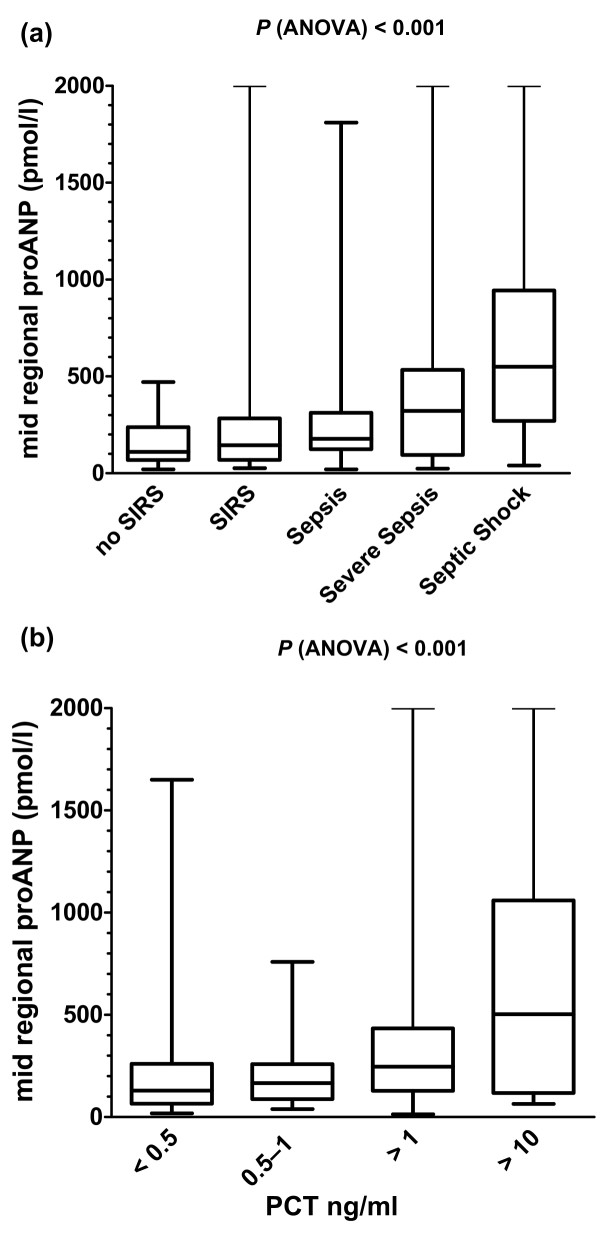
Pro-atrial natriuretic peptide (ANP) according to severity of disease and circulating procalcitonin (PCT) levels. All patient data were grouped according to **(a) **the severity of the disease following consensus criteria ('no SIRS',' SIRS', 'sepsis', 'severe sepsis' and 'septic shock') or **(b) **circulating PCT concentrations. Data from all time points (i.e. on admission, day 2, day of discharge and death) are displayed. Solid lines denote median values, boxes represent 25th to 75th percentiles and whiskers indicate the range. ANOVA, analysis of variance.

Figure 1a shows the distribution of pro-ANP values according to severity of infection (i.e. SIRS, sepsis, severe sepsis and septic shock) and serum PCT concentrations. Depending on the clinical severity of the infection, pro-ANP values exhibited a gradual increase from the group with SIRS to the group with septic shock (*P *< 0.001). Similarly, circulating pro-ANP levels showed a similar gradual increase when categorized based on PCT levels (Fig. 1b).

Post-test analysis revealed a significant difference (*P *< 0.001) between patients without SIRS, SIRS, sepsis and severe sepsis as compared with patients with septic shock. There was no significant difference between patients with severe sepsis and those with septic shock. Accordingly, patients with PCT levels greater than 10 ng/ml and greater than 1 ng/ml had significantly higher pro-ANP levels than did patients with PCT levels of 0.5–1 ng/ml and under 0.5 ng/ml (*P *< 0.001).

Pro-ANP levels correlated with serum IL-6 levels (r = 0.22; *P *< 0.001), and with serum and urine osmolarity (r = 0.55 and r = -0.43, respectively; *P *< 0.001), but not with serum sodium (r = 0.03; not significant) and only weakly with urine sodium concentrations (r = -0.17; *P *< 0.01).

### Pro-atrial natriuretic peptide and outcomes in patients with sepsis, severe sepsis and septic shock

**Figure 2:**
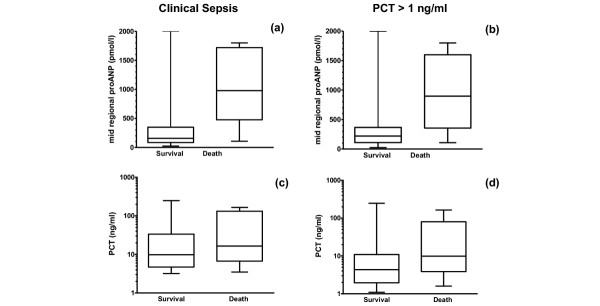
Pro-atrial natriuretic peptide (ANP) and procalcitonin (PCT) levels in surviving as compared with nonsurviving patients. Data from the patients on admission are shown. Patients were grouped **(a, c) **by clinical diagnosis of sepsis according to international guidelines or **(b, d) **by circulating PCT levels in excess of 1 ng/ml. Solid lines denote median values, boxes represent 25th to 75th percentiles and whiskers indicate the range.

Figure 2 shows all pro-ANP values in survivors and nonsurvivors with sepsis, severe sepsis or septic shock, measured during their stay in the ICU. Thereby, patients were grouped by clinical diagnosis of sepsis according to international guidelines (panels a and c) or by circulating PCT level in excess of 1 ng/ml (panels b and d). The median pro-ANP value in the nonsurvivors was significantly greater than in the survivors, independent of grouping used. This difference in pro-ANP values was clear on the first day of admission to the ICU (*P *< 0.001). In contrast, the difference between the survivors and nonsurvivors on the first day of admission was not significant for PCT (*P *= 0.38 and *P *= 0.05, respectively), CRP, or IL-6 (data not shown for CRP and IL-6). Similarly, in patients without infections pro-ANP values were not higher in nonsurvivors than in survivors (all time points: 197.2 ± 361.5 pmol/l versus 226.0 ± 183.4 pmol/l, *P *= 0.7; on admission: 221.5 ± 209.7 pmol/l versus 161.3 ± 132.1 pmol/l, *P *= 0.3).

**Figure 3:**
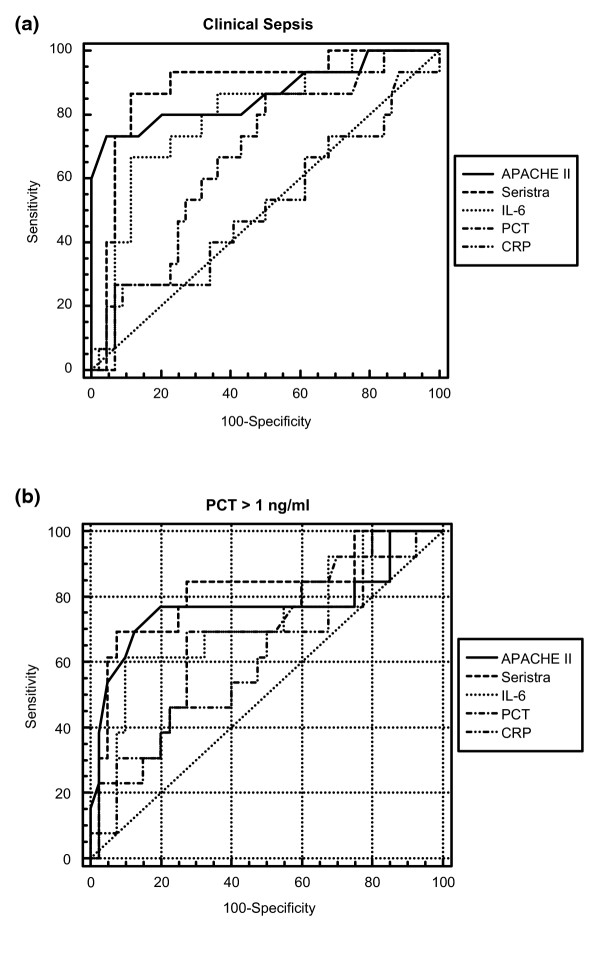
Receiver operating characteristic plot analysis of different biomarkers with respect to outcome prediction of sepsis. Patient data on admission were grouped by **(a) **clinical diagnosis of sepsis according to international guidelines or by **(b) **circulating procalcitonin (PCT) levels in excess of 1 ng/ml. Sensitivity was calculated in nonsurvivors, and specificity in survivors. APACHE, Acute Physiology and Chronic Health Evaluation; CRP, C-reactive protein; PCT, procalcitonin.

To define an optimal decision threshold for pro-ANP values in septic patients, we performed receiver operating characteristic (ROC) plot analysis, including only data from patients with sepsis, severe sepsis, or septic shock obtained within the first 48 hours after admission to the ICU. Sensitivity was calculated among those patients who did not survive sepsis, and specificity was assessed among those patients who were discharged from the ICU. For comparison, the same ROC plot analysis was performed with CRP, PCT, IL-6 and APACHE II score. Table 3 shows the area under the ROC curve (AUC) for all parameters, including the 95% confidence interval. The AUC for pro-ANP was 0.88, which was significantly higher than the AUCs for PCT and CRP, and similar to the AUC for the APACHE II score (0.86). ROC curves are shown in Fig. 3. Again, patients were grouped by clinical diagnosis of sepsis according to international guidelines (panel a) or by circulating PCT levels in excess of 1 ng/ml (panel b), yielding comparable results.

The optimal threshold for pro-ANP was 530 pmol/l. At this cut-off, the sensitivity for correct prediction of death in the ICU was 86.7% and the specificity was 88.6%. Considering a prevalence of 33% for death in the ICU as a result of sepsis, the positive predictive value (PPV) of pro-ANP was 72.2% with a negative predictive value (NPV) of 95.1%. None of CRP, PCT, or IL-6 had similarly high values for sensitivity, specificity, PPV and NPV.

The APACHE II score was also predictive for prognosis but yielded lower values as compared with pro-ANP. At an APACHE II threshold of 30, the sensitivity was 73.3% and the specificity was 95.6% (PPV = 84.6 %, NPV = 91.5 %). At a cut-off of 25, which was recommended by the US Food and Drug Administration for the use of Xigris^®^, sensitivity was 80.0%, specificity 75.6%, PPV 48.0% and NPV 91.4%.

Because PPV and NPV are dependent on the prevalence of the disease, Table 4 shows the relative likelihood with the prevalence independent likelihood ratio for different cut-offs.

## Discussion

ANP and pro-ANP are markers for congestive heart failure [8-10], but their pathophysiological and prognostic significance in severe sepsis and septic shock is not yet understood. In the present study we found a significant increase in mid-regional pro-ANP in the plasma of sepsis patients as compared with patients without sepsis and healthy individuals. This increase was most marked in those patients with sepsis who did not survive their disease. Importantly, on the first day of admission to the ICU, pro-ANP, but not other markers of infection and inflammation such as CRP and PCT, were significantly increased in nonsurvivors as compared with survivors, suggesting that pro-ANP levels represent a new and valuable prognostic tool in patients with sepsis. At a threshold of 530 pmol/l, pro-ANP had a sensitivity of 86.7% for death in the ICU with sepsis, with a specificity of 88.6%; these figures were not reached by any of the other tested biomarkers.

As is generally recommended, we diagnosed sepsis, severe sepsis and septic shock using well defined and widely accepted clinical guidelines [25,26]. However, true gold standards for the diagnosis of infections do not exist, and clinical classification of critically ill patients is not 100% certain despite the use of these guidelines, not only in sepsis trials but also in routine bedside use [27,28]. An ideal sepsis marker should permit early diagnosis, should provide information about the course of disease, and should help to differentiate bacterial from noninfectious and viral causes of systemic inflammation. It was shown that PCT has some of these features and is helpful in diagnosing septic conditions [29-31]. Therefore, we also classified pro-ANP levels according to circulating PCT levels, which are not subject to the uncertainty associated with clinical sepsis definitions. Importantly, the prognostic value of pro-ANP was similar independent of the classification system used, which suggests that our findings are reproducible. Thus, pro-ANP is of prognostic value in critically ill septic patients, in contrast to PCT, which is predominantly a diagnostic parameter.

The first observations that ANP may play a role during endotoxic shock came from animal studies in which ANP was elevated within 2–6 hours after lipopolysaccharide injection [32,33]. Subsequent studies in critically ill humans showed an association of ANP with various cardiac physiological parameters [34,35].

The use of different assays might be responsible for part of the inconsistency in reported findings over recent years. Whereas Berendes and coworkers [14] found no association of ANP values with severity of the disease or mortality in critically ill patients, Hartemink and coworkers [13] found a strong association of ANP levels with myocardial depression in septic shock and with lethal outcome in 14 patients. A similar association of cardiac depression in septic shock was described for NT-pro-ANP in 17 patients [12].

Unfortunately, a limitation of our study is that cardiac indices were not routinely assessed by pulmonary artery catheter. Therefore, the precise mechanisms of pro-ANP release in patients with sepsis remain unknown. Nevertheless, a cardiac origin of natriuretic peptides makes an association with septic cardiac dysfunction likely. In addition, apart from volume overload, osmolarity rather than sodium concentration is associated with pro-ANP release, as suggested by regression analyses in our patients. Based on our findings and recent reports in the literature [36], in critically ill patients increased levels of natriuretic peptide are not specific for decompensated heart failure. In this context, the increase in ANP levels in septic shock may be potentiated by IL-6 elevation [15]. A recent study in meningococcal sepsis provided conclusive evidence that IL-6 is directly involved in myocardial depression [37]. Accordingly, in the present study IL-6 levels were correlated with pro-ANP levels, albeit relatively weakly. IL-6 had lower value in terms of outcome prediction than did mid-regional pro-ANP, which may be due to differences in the half-life of the molecules. The half-lives of both IL-6 and mature ANP are short, and measurement of those markers in septic patients does not allow a direct conclusion to be drawn regarding the level of production. We recently developed a sandwich immunoassay for the detection of a mid-regional fraction of pro-ANP in plasma [20]. This fragment has a much longer half-life in plasma, and because it is produced in equimolar concentrations to the mature hormone, it mirrors true production of ANP. Furthermore, it is possible that mid-regional pro-ANP exerts a physiological effect on its own, as is described for other fragments of NT-pro-ANP [38] and fragments of other prohormones, such as pro-adrenomedullin amino-terminal 20 peptide [39].

Measurement for ANP or fragments of NT-pro-ANP is potentially influenced by other factors, such as sex, age and kidney function, as is discussed elsewhere for brain-type natriuretic peptides [40,41]. Indeed, we observed a significant correlation of circulating pro-ANP levels with serum osmolarity and creatinine. Measurements in nonseptic patients with kidney failure revealed mostly normal pro-ANP values, and it is therefore possible that the observed elevation in pro-ANP and creatinine in this study is a result of kidney failure related to sepsis.

Sepsis is a complex syndrome, and the immunological and biochemical situation may vary considerably between individual patients [3,4]. In the past almost all intervention trials failed to show any benefit from therapy for sepsis, and sepsis intervention has been termed the 'graveyard for pharmaceutical companies' [7,42]. Reasons for this may be found in immunological heterogeneity and insufficient patient stratification in those trials [5]. The need for markers that permit better stratification of patients with different stages of sepsis is underlined by the ongoing discussion concerning recombinant human activated protein C (drotrecogin alpha; Xigris^®^) [42-45]. The US Food and Drug Administration approved recombinant human activated protein C only for those patients with an APACHE II score in excess of 24, and thus only for those patients with the greatest risk for dying [28,46,47]. The APACHE II score – a complex algorithm – was not originally developed for individual outcome prediction in sepsis patients [48]. Despite its limitations, outcome predictors such as the extensively evaluated APACHE II score are helpful in identifying those septic patients who are at high risk for death and who are more likely to benefit from intervention [6]. In the present study the prognostic value of pro-ANP levels was comparable to that of APACHE II score. Importantly, mid-regional pro-ANP it is easier to determine than a physiological score and mirrors distinct pathophysiological changes that occur in sepsis.

## Conclusion

In septic patients, we found that APACHE II score and mid-regional pro-ANP level on admission to a medical ICU had similar ability to predict outcome. The results of our study are novel and of interest because they may help to improve stratification of septic patients. Our findings are descriptive in nature and warrant validation in future prospective studies, including measurement of cardiac indices or evaluating patients who have undergone surgery. If our findings are confirmed, then mid-regional pro-ANP might become a new and useful additional prognostic marker for individual risk assessment in sepsis, and may represent a helpful tool for patient stratification in future intervention trials.

## Key messages

• 	In septic patients mid-regional pro-ANP levels on admission to a medical ICU had a similar ability to predict outcome as did the APACHE II score.

• 	Pro-ANP levels appear to be a useful tool for individual risk assessment in septic patients and for stratification of high risk patients in future intervention trials.

• 	Because our findings are descriptive in nature, further prospective studies are warranted to validate our results.

## Abbreviations

ANP = atrial natriuretic peptide; APACHE = Acute Physiology and Chronic Health Evaluation; AUC = area under the curve; CRP = C-reactive protein; ICU = intensive care unit; IL = interleukin; NPV = negative predictive value; NT = amino terminal; PCT = procalcitonin; PPV = positive predictive value; ROC = receiver operating characteristic; SIRS = systemic inflammatory resonse syndrome.

## Competing interests

NG, JS and AB are employees of BRAHMS AG, the manufacturer of the pro-ANP assay (BRAHMS Seristra^® ^LIA; BRAHMS AG, Hennigsdorf/Berlin, Germany). BM has served as a consultant and received payments from BRAHMS AG to attend meetings related to the trial and for travel expenses, speaking engagements and research.

## Authors' contributions

BM conceived the study, collected the data, drafted the protocol and supervised the writing of the manuscript. NGM, JS and AB were involved in assay development. NGM and MCC conducted statistical analyses and wrote the report. All authors read and approved the final manuscript.

## Figures and Tables

**Table 1:** Clinical diagnoses of the patients

Diagnosis	Details	Number of patients^a^
Respiratory	Pneumonia (33), chronic obstructive pulmonary disease (14), acute asthma (3), bronchial carcinoma (3), pneumothorax (3), pharyngeal obstruction (2), toxic pulmonary oedema (2), Wegener's granulomatosis (1)	61
Cardiovascular	Myocardial infarction (12), heart failure (11), pulmonary embolism (2) haemorrhagic shock (1)	26
Abdominal	Gastrointestinal bleeding (7), abdominal infection (6), urinary tract infection (5), acute renal failure (3), hepatic coma (3)	24
Cerebral	Ischaemic stroke (5), subarachnoid (4) or intracerebral (3) haemorrhage, seizures (3), suicidal intoxication with sedatives (5), cavernous sinus thrombosis (1)	21
Others	Leukaemia (7), postoperative (6), diabetic coma (3), other infections (3)	19

^a^One patient can have more than one diagnosis, and so the total exceeds the absolute number of patients (*n *= 101).

**Table 2:** Site of infection and microbiology

Site of infection	Details	Number of patients^a^
Lung	*Streptococcus pneumoniae *(6), *Pseudomonas aeruginosa *(5), *Haemophilus influenzae *(3), *Streptococcus pyogenes *(3), *Staphylococcus aureus *(3), *Klebsiella pneumoniae *(2), *Escherichia coli *(2), *Enterobacter *spp. (2), *Streptococcus salivarius *(1), *Legionella pneumophilia *(1), unknown (16)	44
Urinary tract	*Escherichia coli *(5), *Pseudomonas aeruginosa *(1)	6
Abdominal (gastrointestinal tract, liver, bile duct, and pancreas)	*Clostridium difficile*-associated colitis (1), unknown (4)	5
Others	Meningococcal meningitis (1), sepsis with *Torulopsis glabrata *(1), malaria with *Plasmodium falciparum *(1)	3

^a^An infection was diagnosed in 58% of the patients (on admission in 53 patients; five additional patients developed sepsis during their stay in the medical intensive care unit).

**Table 3:** Area under the curve of receiver operating characteristic plot analysis

Parameter	AUC	95% CI	*P *(versus pro-ANP)
Pro-ANP	0.88	0.77–0.95	-
APACHE II	0.86	0.74–0.93	0.79
IL-6	0.79	0.66–0.88	0.34
PCT	0.67	0.53–0.78	0.027
CRP	0.51	0.38–0.64	< 0.001

ANP, atrial natriuretic peptide; APACHE, Acute Physiology and Chronic Health Evaluation; AUC, area under the curve; CI, confidence interval; CRP, C-reactive protein; PCT, procalcitonin.

**Table 4:** Sensitivity, specificity, positive likelihood ratio, negative likelihood ratio and odds ratio at different cut-off levels of pro-ANP

Pro-ANP cut-off (pmol/l)	Sensitivity (95% CI)	Specificity (95% CI)	LR^+^	LR^-^	Odds ratio (95% CI)
350	93% (68–99%)	77% (62–88%)	4.1	0.09	47.6 (5.5–408)
530	86% (59–98%)	88% (75–96%)	7.6	0.15	50.7 (8.7–293)
700	73% (44–92%)	93% (81–98%)	10.7	0.29	37.5 (7.3–193)

ANP, atrial natriuretic peptide; CI, confidence interval; LR^-^, negative likelihood ratio; LR^+^, positive likelihood ratio;
